# Rhyme and Word Placement in Storybooks Support High-Level Verb Mapping in 3- to 5-Year-Olds

**DOI:** 10.3389/fpsyg.2018.00889

**Published:** 2018-06-05

**Authors:** Kirsten Read, Jacqueline Quirke

**Affiliations:** Department of Psychology, Santa Clara University, Santa Clara, CA, United States

**Keywords:** high-level vocabulary, word learning, rhyme, shared reading, preschoolers

## Abstract

High-level verbs can be especially challenging for young children to initially map to meaning. This study manipulated the format of a storybook designed to support such verb learning from shared reading. We tested whether 3- to 5-year-olds (*n* = 38) could remember the referents of eight new verbs when presented as essential actions within a narrative story but with differences in placement. Children were randomly assigned to either a rhymed condition, in which target verbs were heard at the end of rhyming stanzas making them maximally appreciable, or a control condition, where the verbs were presented in the same story, but not in final position or within a rhymed stanza. After hearing the story, each child was given three sets of retention questions testing their identification, demonstration, and production of the target verbs. Children identified and successfully demonstrated more target verbs in the rhymed condition than the control condition, and only in the rhymed condition did children’s initial verb mappings exceed chance. No differences between conditions were found in children’s ability to produce the target verbs, in part because of how often they reverted to more generic terms to describe the actions in the story. Nonetheless, these findings support the hypothesis that giving children maximal support within a storybook reading context can facilitate an initial grasp on challenging verbs.

## Introduction

Shared reading is a common activity at home and in the preschool classroom that provides rich support for language learning, and for preschool-aged children it is a sure way to help build and diversify their vocabulary (e.g., Ninio, 1983; Elley, 1989; DeBaryshe, 1993; Sénéchal and Cornell, 1993; Robbins and Ehri, 1994; DeTemple and Snow, 2003; Farrant and Zubrick, 2013). Supporting children’s vocabulary growth in the preschool years is especially important because vocabulary size at school entry predicts a host of other language, literacy, and academic outcomes (e.g., Storch and Whitehurst, 2002; Dickinson et al., 2003, 2010). But, not all vocabulary is equal. It may be especially important to support children’s learning of “high-level” words beyond those typical for a preschool-aged child to hear in everyday conversation, in order to bolster their language development (e.g., Weizman and Snow, 2001; Houston-Price et al., 2014).

High-level words are infrequent by nature and often challenging to apprehend, and so shared reading is an especially good source for high-level vocabulary learning because it can provide a supportive narrative context. The new words are connected to a story, not just presented in isolation, and thus more semantic connections between new words and known content words can be established (e.g., Chilton and Ehri, 2015). Children may stay more engaged and interested in understanding the meanings of new words that are part of a story. Also, stories present more diverse vocabulary than everyday conversation (Montag et al., 2015), so it is not unusual for children to hear a word for the first time in a storybook context.

Research has shown that giving young children simple explanations or explicit definitions of new challenging words (e.g., Beck and McKeown, 2007; Wilkinson and Houston-Price, 2013; Houston-Price et al., 2014) or asking questions about new words when they are encountered in a story (e.g., Ewers and Brownson, 1999; Ard and Beverly, 2004) can help build vocabulary, especially when the story and explanations are heard repeatedly. On the other hand, research has also shown that after even just a single encounter within a story without any explicit elaboration, children can begin to build a representation of what a novel word refers to (e.g., Elley, 1989; McLeod and McDade, 2011; Read, 2014). And, in fact, children may often have to pick up new vocabulary incidentally rather than explicitly during shared reading – In a recent study of parents naturally reading a book with their 6-year-olds that contained 38 high-level words, it was very rare that parents stopped to explain or even comment on these words without the children asking for clarification, and especially infrequent if the words were not easy to define, or in a page-final position within the text (Evans et al., 2011).

While children as young as three are frequently able to “fast map” or make an initial hypothesis about the meaning after just the first encounter with a new word (e.g., Carey and Bartlett, 1978), there are still some words that are more challenging than others. English-speaking children appear to have more difficulty fast mapping verbs in comparison with other categories of words such as nouns and adjectives (e.g., Rice et al., 1990; McLeod and McDade, 2011). McLeod and McDade (2011) most recently found that if preschool-aged children were given five novel nouns and five novel verbs within a story read three times, children could correctly map at least some of those new words in a simple four-alternative picture choice task afterwards. However, similar to previous studies, children were much better at identifying the new nouns’ referents than depictions of the novel verbs. In fact, children’s correct responses to the novel verbs did not appear to rise above chance performance in this simple mapping task. Thus many verbs just by their nature are challenging high-level words.

It is no surprise that verbs tend to be more challenging to learn from storybooks than nouns for several reasons. In English (and other languages), objects are labeled or mentioned by adult readers more often than actions, creating a noun-bias in the language environment in the context of shared reading (e.g., Tardif et al., 1999; Choi, 2000; Murase, 2014). The referents of verbs tend to be less concrete compared to nouns often making them harder to illustrate in the storybook context. In addition, verbs are given less phonological stress, due to their inconvenient tendency to appear in a sentence-medial position instead of at the beginning or the end, where they would naturally receive verbal highlighting. In natural settings, adult readers are more likely to elaborate on challenging words when they occur at the end of the page, instead of in the middle of a line (Evans et al., 2011), but verbs are not nearly as likely as nouns to appear in this location (Clark, 2010).

While previous research on learning new vocabulary items from storybooks has had a lopsided focus on nouns, one study by Brackenbury and Fey (2003) demonstrated some successful incidental verb learning in storybook contexts. They found that 4-year-old children were able to demonstrate quick incidental learning of challenging manner-of-motion verbs like *saunter* or *trudge*, rapidly retaining these new words after hearing them in a single story (though repeated 13 times). In this study, the children may not have fully understood each verb’s meaning within the story or been able to accurately generalize the new verbs to new situations, but they were able to identify an illustration of the newly learned verb above the level of chance. At the very minimum, young children may be able to connect a new, challenging verb to a picture depicting its referent.

While this finding is promising, it is still not clear whether young children can incidentally fast-map some basic referential information about high-level verbs from more natural shared reading experiences in which those verbs are not explicitly highlighted, defined, or excessively repeated. In the present study, we tried to maintain the integrity of a typical shared reading experience for preschool-aged children, while attempting to support challenging high-level verb learning in several ways.

First, while novel nouns in storybooks are often accompanied with a corresponding illustration of a referent, challenging verbs are not as frequently depicted with pictures meant to demonstrate their meanings (e.g., Beck and McKeown, 2007; Houston-Price et al., 2014). Thus, in our study, we intentionally illustrated the action of each target verb with hand drawn pictures in order to offer children a clear referent.

Research with older children has found that it may be easier to learn new words when they are set in a narrative context, than simply presented in isolation (e.g., Chilton and Ehri, 2015), but studies that have focused on (or included) verb learning from stories read with young children, sometimes take away the potential usefulness of the narrative by using unrealistic actions, stripping the new verbs of purpose. For example, McLeod and McDade (2011) deliberately used novel one-syllable words for actions that would not have synonyms in real English, e.g., *gis* meant “suspended upside down in the air with legs wide apart,” or while Brackenbury and Fey (2003) gave children real verbs like *saunter*, they were presented in contexts where there was no particular motivation to use that particular verb and a simpler synonym like *go* or *walk* could easily serve as a replacement. However, we hypothesized that motivating the particular action described by each target verb in our story, even if it did have a simpler synonym, may help make the use of those verbs more realistic and also more central within the narrative. For example, in the story, Kara needed to really *peer* at the map in order to know which way to go to find the treasure, and she needed to *cast* some light into the cave with her flashlight in order to be able to go in and find the treasure – every verb used was part of the sequence of actions that were necessary to help the protagonist reach her end goal.

In addition, recent work has found that small differences in where new vocabulary appears and how it is presented within a story can affect how well it is apprehended. For example, when novel target words appear in rhyming stanzas 2- to 5-year-olds are better at remembering the words (Johnson and Hayes, 1987; Read et al., 2014; Király et al., 2017), as well as mapping those words to new referents (Read, 2014), perhaps in part because rhymes encourage verbatim recall that highlights the specific words rather than only the gist of the meaning (e.g., Johnson and Hayes, 1987). In addition to the support that rhyme scheme may lend, placing new words at the end of a sentence or rhyme in particular and, in a storybook context, before a page break can add even a moment of helpful emphasis (e.g., Read, 2014) and an opportunity to pause and reflect on the word (e.g., Evans et al., 2011). Hearing verbs at the ends of sentences is not the most common placement for them, but it’s also not impossible to put them there, and so we devised rhyming stanzas that could end with our target high-level verbs to maximize their chance of being noticed and remembered by preschool-aged children.

Given that verb learning is more challenging but can be so beneficial for young language learners, we tested whether the fine-grained manipulations to storybook format that appear to boost novel noun learning can also support initial learning of more challenging verbs. Research on how children apprehend novel nouns from storybooks has indicated the usefulness of rhyme, sentence, and page final location, as well as integration into the story narrative to aid children in learning object words. Since storybooks often lack these key aspects for verb learning, we tested their effects on challenging verbs. In the current study, we did not control for integration. In both conditions, verbs were integrated into the narrative, creating a fluid story. However, we manipulated rhyme and location together. The following were the two main research hypotheses that we tested:

(1) Children can fast map challenging high-level verbs from a storybook after just one exposure within the story if those words are clearly depicted and central to the story.
(2) Placement of the challenging verbs at the end of rhyming stanzas will enhance children’s ability to map those verbs.

## Materials and Methods

### Participants

There were a total of 38 participants between the ages of 3 to 5 years (*M* = 52 months) who participated in this study. One participant’s data was excluded due to inattentiveness and an inability to complete the questions. All the participants were randomly assigned to one of two conditions. In the rhymed, experimental condition, there were a total of 19 participants (10 girls, and 9 boys) ranging in age from 35 to 65 months (*M* = 51.5 months). In the unrhymed, control group, there were 18 participants (7 girls and 11 boys) ranging in age from 36 to 62 months (*M* = 51.7 months). The entire group of participants came from two nearby preschools serving an educated, middle income and ethnically diverse population. Each participant spoke English as a first language and was very familiar with shared storybook reading as a daily activity in the classroom as well as at home. Written informed consent was given by a parent of each child before they were invited to participate.

### Materials

**FIGURE 1:**
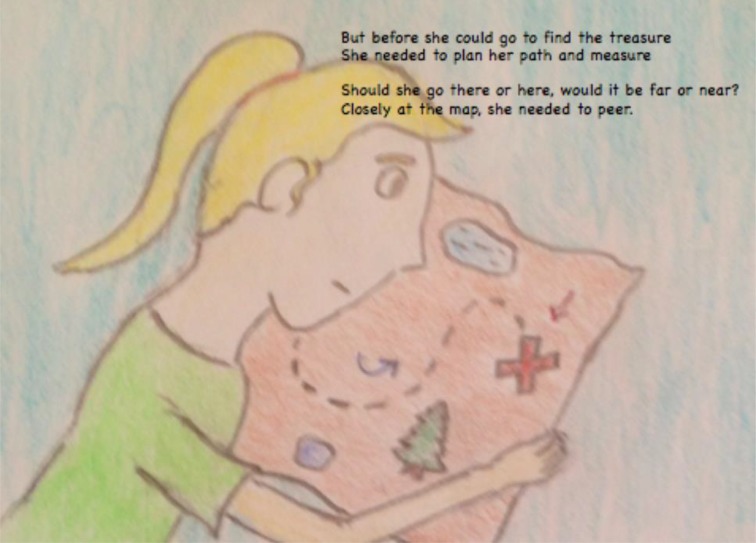
Example page from the storybook with text from the rhyme condition.

For the purposes of this study, we wrote two versions of a story we called *Kara’s Treasure Hunt* created specifically for teaching eight challenging verbs for children this age – *peer, depart, clamber, ascend, cast, demolish, extract*, and *applaud*, and an undergraduate art student volunteered to create illustrations for the book with drawings intended to clearly depict each action (see **Figure 1** for example page). Real verbs were chosen instead of novel words in order to incorporate realistic actions depicted with actual words with natural phonotactic structure. The target verbs were chosen by selecting a few sources and narrowing down potential verbs. We avoided verbs listed on the MacArthur-Bates Communicative Development Inventory (MCDI) (Fenson et al., 2007) and those on the Peabody Picture Vocabulary Test (PPVT-4) (Dunn and Dunn, 2007) for children under 10 years of age, in order to ensure that the target words were not likely to be familiar for 3- to 5-year-olds. Additionally, we chose target verbs used in previous research on high-level vocabulary (e.g., *depart* and *applaud* were used in Houston-Price et al., 2014), which could easily be depicted in illustration, were plausible within the narrative of the story and were rhymable for the purposes of the book.

In addition, we created a short verb checklist for parents. Parents indicated whether they believed their child “understands” or “says” each word on a list of 20 verbs taken from within the story (e.g., *smiling, waving, and hurrying*) including the eight target high-level verbs. No parents reported that their child used any of the target verbs, and only on two occasions did a parent report that they believed their child might understand any of the target verbs (one parent marked *demolish*, and another marked *applaud*). This was taken as evidence that the targets were, indeed, high-level and unfamiliar to our sample of children.

The story read to each child was one variation (based on condition) of a narrative about a little girl who goes on a treasure hunt. Each step of her treasure hunt incorporates one of the target verbs, and is illustrated on a single page to support mapping the word to the depiction (e.g., Flack and Horst, 2017). The children either heard the story with the target verbs presented at the end of rhyming stanzas making them maximally predictable and memorable (e.g., Read, 2014), or heard a control version of the story in which the same verbs were presented, but not in final position or within a rhymed stanza. **Figure 1** depicts an example of a page from the rhymed version in which children heard the accompanying text: *But before she could go to find the treasure/She needed to plan her path and measure/Should she go there or here, would it be far or near?/Closely at the map, she needed to* ***peer***. In the unrhymed condition, participants saw the same illustration with the nearly identical, but unrhymed text: *But before she could go to find the treasure/She needed to measure and plan her path/Should she go here or there? Would it be near or far?/Kara needed to* ***peer*** *closely at the map.*

The books were the same length. Both were nine pages of content with 30–36 words per page (*M* = 33 words per page), similar to those used in previous research from our lab. Children in this age range demonstrated in pilot testing that they understood and enjoyed the stories.

### Procedure

Both conditions had an identical procedure. The researcher approached each participant individually during morning free play time in their preschool classroom, told him or her about the task, and asked whether or not they wanted to hear a story book. If they assented, they were led to a quiet room or corner of a classroom. Depending on which condition the child had been randomly assigned to, the researcher either read the rhymed or the unrhymed version of the story, reading every page aloud, keeping the book and its pictures in the participant’s line of sight. The story was read once by the experimenter in a child-friendly, appropriately paced way, without extra commentary. If a child made a comment or asked a question during the story reading, the experimenter replied minimally with “mmhmm” or “let’s see what happens next” and did not repeat or define any of the target words.

There were three measures which directly followed the story. Each set of questions included a warm-up question, using the verb *napping*. This ensured that the child would become accustomed to the type of question before being introduced to the questions using the target verbs. The first set, the identification questions, involved a simple multiple-choice picture pointing task in which a child would be asked, e.g., “Which picture shows *peering*” with three pictures to choose from (randomized, with location of the target picture counterbalanced across questions). The target and distracter pictures for this task were cropped versions of the illustrations that children had seen previously within the story, thus each depiction served as a target once and a distracter twice within testing in both conditions. Children were asked about each of the eight target verbs. Coders blind to condition assigned one point for each correct, matching picture that a child pointed to yielding an identification score for each child that could range from 0 to 8. The second set of questions, demonstration questions, asked the child to act out each target action, e.g., “Can *you* show me *peering?*” Children were encouraged to demonstrate using their own bodies or hands, and coders blind to condition assigned a point for each demonstration that captured the main action of the verb (e.g., waving goodbye to show *departing*, lifting knees to show *clambering*, or banging a fist to show *demolishing*) or half points for actions that partially depicted the actions of the verb (e.g., walking with fingers but without an upward direction to show *ascending*, or reaching with the whole hand without a careful manner to show *extracting*). Thus each child received a demonstration score that could range from 0 to 8. Finally, the third set, the production questions, showed a picture from the story of an action, and asked the child “What is she doing here?” All of the children’s answers were recorded for each of the eight pictures they were shown, but coders blind to condition only assigned a point when the child said the target verb in each case yielding possible production scores of 0–8 for each child. The stories and questions were presented to the child in a natural child-friendly manner and no corrective feedback, only positive encouragement was provided (e.g., “good choice, let’s do the next one!” regardless of whether a child answered a question correctly).

Children’s responses were all hand-recorded by the initial researcher and then two additional researchers double-scored the responses. Reliability between the two scorers was 100% for the identification questions, 97% for the demonstration questions, and 97% for the production questions.

## Results

**FIGURE 2:**
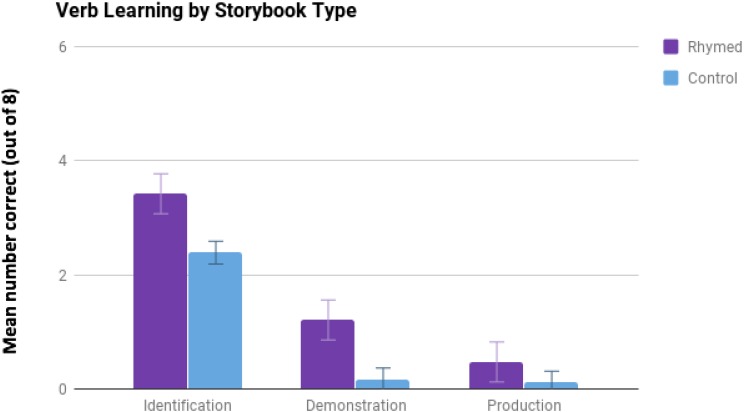
Mean correct scores for children in each test by condition. Error bars represent standard error of the mean for each measure.

In order to test whether children were more successful at fast-mapping challenging high-level verbs in the rhyming condition than in the control condition, we analyzed responses comparing the two groups based on mean number of correct responses for children in each test type in each condition with independent samples *t*-tests (see **Figure 2**).

### Identification

In the three-alternative choice identification task, children’s scores were normally distributed without outliers in both conditions, and did not differ between conditions in variability. When comparing children’s correct identifications of the picture that corresponded to each target verb from the story in the three alternative choice picture pointing task, we found that children in the rhyming condition correctly identified more challenging verbs (*M* = 3.42, *SD* = 1.54) than children in the control condition (*M* = 2.39, *SD* = 1.04), *t*(35) = 2.38, *p* = 0.023, with an effect size of *d* = 0.79. In addition, when we compared each group of children’s mean correct identifications to chance level performance (2.667 or one out of three correct over eight trials), only those children in the rhyming condition made more correct identifications than would be expected by chance, *t*(18) = 2.14, *p* = 0.047, while correct identifications in the control condition did not differ from chance, *t*(17) = -1.14, *p* = 0.271.

### Demonstration

When comparing children’s ability to physically demonstrate the target verbs that they had heard in the story, we found that children in the rhyming condition correctly (or closely) approximated the actions of more target verbs (*M* = 1.21, *SD* = 1.36) than children in the control condition (*M* = 0.17*, SD* = 0.34). Despite non-homogeneous variability between the two conditions, this difference was found to be significant with a non-parametric Mann–Whitney *U* test, *p* = 0.006, and had a large effect size of *d* = 1.05. Also, Wilcoxon Signed Rank tests showed that in this challenging task only the children in the rhyme condition produced significantly more than zero demonstrations of the verbs, *p* = 0.007, while children in the control condition did not, on average, differ from zero in how often they could closely demonstrate the action of the target verbs, *p* = 0.386.

### Production

In the most challenging test of children’s fast-mapping of these new verbs, we also found that children tended to produce more target verbs when asked what was depicted in the illustration in the rhyme condition (*M* = 0.53, *SD* = 0.84) than in the control condition (*M* = 0.11, *SD* = 0.47). However, even when correcting for non-homogeneity of variance using a non-parametric Mann–Whitney *U* test, this difference was not significant, *p* = 0.118, in part due to a floor effect, though there was still a moderate effect size, *d* = 0.53. In addition, when we compared children’s median verb productions in each condition to zero using Wilcoxon Signed Rank tests, again only those children in the rhyming condition made significantly more than zero correct productions, *p* = 0.001, while correct productions in the control condition did not differ from zero, *p* = 0.480.

Taken together, the results of these three tests show that children comprehended the actions within the story, and also that there was consistent advantage for children in the rhyming condition in remembering and mapping these very challenging new verbs, heard only once in the story.

## Discussion

The main finding from this study was that even very challenging vocabulary can be supported at an initial stage of learning through shared storybook reading by highlighting new words through end-of-line placement and rhyme. The task of fast mapping eight high-level verbs after only a single incidental exposure within a story is typically challenging for children in this age range despite their “sponge-like” word learning capacity. In both conditions there were supports in place for word comprehension that went beyond previous research – we purposefully created a narrative that made the new words relevant (e.g., Chilton and Ehri, 2015), included only a single illustration on each page (e.g., Flack and Horst, 2017), and tried to clearly depict the actions referenced by the target high-level verbs. With respect to our initial hypothesis, we did *not* find that children could fast-map high-level verbs after a single exposure within a story when those verbs were clearly depicted and central to the story – these features alone were not enough for children in the control condition to show above chance performance after a single reading of the stories. It was only with those features *plus* the rhyme and sentence final placement of target words in the test condition (e.g., Read, 2014) that children were able to achieve above chance performance, hence the significant differences between conditions in our measures. And so, with respect to our second hypothesis, we did find that placement of the challenging verbs at the end of rhymed stanzas *in addition to* depicting them clearly and making them central to the narrative of the story enhanced children’s ability to map those verbs.

**FIGURE 3:**
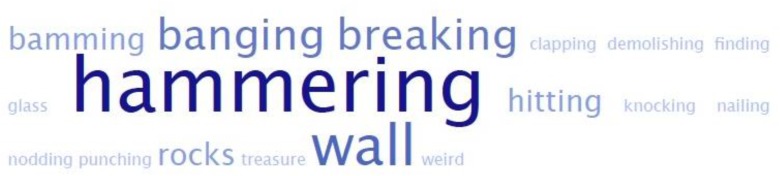
Word cloud visualization for children’s responses (*n* = 33) across both conditions in the production test to the item *demolish*. Words that appear larger in the word cloud were higher frequency responses.

These findings are an exciting demonstration of the power of subtle changes in storybook format that can have a measurable impact on challenging word learning. However, it is important to be clear what kind of “learning” this actually demonstrates. First, because we deliberately used real English high-level verbs in this study (similar to Robbins and Ehri, 1994; Brackenbury and Fey, 2003; Houston-Price et al., 2014) children were likely to already know simpler synonyms for the target words (e.g., *peer* vs. *look at*, or *depart* vs. *go*). Because of this the task of learning the new word may be simpler – as others have noted, a child faced with a new synonym is learning a new label for a concept they already understand, rather than learning a whole new concept (e.g., Sénéchal and Cornell, 1993; Brackenbury and Fey, 2003). In part, this may be why presenting eight challenging words was not overwhelming to the participants in our study, and why in the production test, while it was hard for them to give the exact target word, children still demonstrated that they comprehended the actions of the story that were depicted by producing near synonyms when asked “What is she doing here?” 86% of the time in the rhyme condition, and 78% of the time in the control condition (e.g., **Figure 3**).

A second important point about learning these new vocabulary items is that the current findings only show an improvement in immediate surface-level mapping of an illustration to the verb that was used to label it. While this is an important first step in learning a new word – simply remembering and identifying its referent, we do not know after just one interaction and testing directly afterwards with the story we created for this study whether there may be any long-term retention or generalization from the initial mappings that children made. This study has only scratched the surface of what processes must underlie children’s fuller retention and understanding of new vocabulary. Further research will look more closely at some of the following important questions:

First, could children show longer-term retention of these verb-referent mappings over time (e.g., 24 h later, 1 week later, etc.)? Other recent research on how well children can pick up words from storybook reading context has shown, in some cases, an improvement in simple identification over time and with repeated testing (e.g., Williams and Horst, 2014) though this has not been tested with high-level verbs or with the support of rhyme and placement cues.

Second, would children show greater benefits or stronger verb-referent mappings if they were read the stories repeatedly? Again, this has been shown empirically to improve children’s novel word identification in shared reading contexts (e.g., Horst, 2013) and is also common in children’s real world experience of storybook reading – most children request and enjoy hearing the same story read multiple times, even sometimes in the same sitting. Thus, we would be interested to know whether there could there be a positive compounding effect for learning challenging high-level verbs after, e.g., three repetitions of a carefully formatted story, or after hearing the storybook each day for a week, etc.

Third, could children begin generalizing their knowledge about these challenging verbs, especially with added repetition or time? In two out of three of our tests of children’s initial mapping of the verbs, they were shown the same illustrations that were in the story, with the same actor performing the action with the same instrument in the same manner for the same purpose, thus no generalization was required. When we asked children to demonstrate the verbs using their own bodies, some generalization was required because of the change in actor, but still children who succeeded in this task appeared to simply mimic what they had just seen depicted in the story. However, further testing could push children’s recognition of the new verbs by changing the actors and the situational contexts in which the verbs are used (e.g., Brackenbury and Fey, 2003) in order to understand how deep a child’s understanding of the new words may go.

Despite these important further questions, the research presented here already shows the potential of paying close attention to the features of storybooks that can be especially supportive for novel high-level word learning. While clearly there are other goals of sharing books with young children alongside teaching vocabulary, the formatting choices that supported fast mapping here were not incompatible with maintaining children’s interest or comprehension of the story in a more general way. It is interesting, in fact, to note that even though the rhymed version of the story sometimes required contorting the syntax in order to get the verbs out to the end of the line and adhere to the rhyme structure (e.g., *closely at the map she had to peer* vs. *she had to peer closely at the map*), the benefits of emphasis that this produced seem to outweigh the costs of infrequent syntactic constructions for helping children map these challenging verbs. In the present study we conflated the target words’ end-line positioning with their phonological predictability based on rhyme. Either of these features alone could have made the target words more attention-getting for children, however, we intentionally combined them in order to “layer on” the potential help that such features could provide children. In the present study, therefore, we cannot parse out the individual effects of rhyme and placement, though previous research has attempted to disentangle the two (e.g., Read, 2014). Read (2014) found that children remembered and identified more novel words when they were rhymed vs. unrhymed, but also when they were the last word in a stanza, rather than the last word in a line at the beginning of a stanza. We capitalized on this finding to give children the best-case scenario for hearing rhymed target words in a maximally attention-getting location, as they are likely to do in typical everyday experiences of shared reading, where the rhymed words are usually at the end of a line.

The final question this work raises is whether the small changes in rhyme and placement that appear to give a boost to novel vocabulary in these stories when read (in a natural way) by experimenters might elicit different kinds of interaction from caregivers when reading the books with children in regular everyday settings. For example, would parents or teachers be prompted to add to the benefits by commenting more often on novel high-level verbs when they are at the ends of stanzas, when they are essential to the unfolding action of the story, or when they fit the rhyme scheme? Would adult readers attempt more definitions and examples for children given one book type over the other, and if so, could that also compound the positive effects of seemingly small changes in how a storybook can frame novel words?

In sum, this study adds to our growing understanding of the dynamic process of word learning for preschool-aged children. Ultimately, situating new vocabulary in relevant, narrative contexts with illustrations that clearly depict the gist of verb meanings is plainly helpful to a child trying to understand those new words. But, in addition to *contextualizing* new vocabulary, *emphasizing* it through strategic placement at the end of a rhyming stanza, before a page turn in a story gives the extra boost needed for children to begin the quick incidental verb learning that they can do so readily with other types of words.

## Ethics Statement

This study was carried out in accordance with the recommendations of the Santa Clara University Office of Research Compliance and the Human Subjects Committee of the Santa Clara University Institutional Review Board (IRB) with written informed consent from a legal guardian of all subjects, and verbal assent of all subjects. All subjects’ parent or legal guardian gave written informed consent in accordance with the Declaration of Helsinki. The protocol was approved by the University of Santa Clara Institutional Review Board.

## Author Contributions

KR was the primary investigator in this research and the lead author of this manuscript. JQ was the undergraduate research team leader in designing and conducting the research and helped to edit the manuscript.

## Conflict of Interest Statement

The authors declare that the research was conducted in the absence of any commercial or financial relationships that could be construed as a potential conflict of interest.
